# Constraint of gene expression by the chromatin remodelling protein CHD4 facilitates lineage specification

**DOI:** 10.1242/dev.125450

**Published:** 2015-08-01

**Authors:** Aoife O'Shaughnessy-Kirwan, Jason Signolet, Ita Costello, Sarah Gharbi, Brian Hendrich

**Affiliations:** 1 Wellcome Trust-Medical Research Council Stem Cell Institute, University of Cambridge, Tennis Court Road, Cambridge CB2 1QR, UK; 2 Department of Biochemistry, University of Cambridge, Tennis Court Road, Cambridge CB2 1QR, UK; 3 Institute for Stem Cell Research, University of Edinburgh, Edinburgh EH9 3JQ, UK; 4 Present address: Sir William Dunn School of Pathology, University of Oxford, Oxford OX1 3RE, UK

**Keywords:** Lineage commitment, Chromatin remodelling, Blastocyst, Transcription, Trophectoderm, CHD4

## Abstract

Chromatin remodelling proteins are essential for different aspects of metazoan biology, yet functional details of why these proteins are important are lacking. Although it is possible to describe the biochemistry of how they remodel chromatin, their chromatin-binding profiles in cell lines, and gene expression changes upon loss of a given protein, in very few cases can this easily translate into an understanding of how the function of that protein actually influences a developmental process. Here, we investigate how the chromatin remodelling protein CHD4 facilitates the first lineage decision in mammalian embryogenesis. Embryos lacking CHD4 can form a morphologically normal early blastocyst, but are unable to successfully complete the first lineage decision and form functional trophectoderm (TE). In the absence of a functional TE, *Chd4* mutant blastocysts do not implant and are hence not viable. By measuring transcript levels in single cells from early embryos, we show that CHD4 influences the frequency at which unspecified cells in preimplantation stage embryos express lineage markers prior to the execution of this first lineage decision. In the absence of CHD4, this frequency is increased in 16-cell embryos, and by the blastocyst stage cells fail to properly adopt a TE gene expression programme. We propose that CHD4 allows cells to undertake lineage commitment *in vivo* by modulating the frequency with which lineage-specification genes are expressed. This provides novel insight into both how lineage decisions are made in mammalian cells, and how a chromatin remodelling protein functions to facilitate lineage commitment.

## Abstract (precis)

**Highlighted article:** The chromatin remodelling protein CHD4 dampens transcriptional priming in totipotent cells, facilitating TE specification during murine embryogenesis.

## INTRODUCTION

The first morphologically distinguishable lineage division during mammalian embryogenesis occurs when totipotent cells of cleavage stage embryos form either the inner cell mass (ICM), which generates the pluripotent cells that will go on to form the embryo proper; or the trophectoderm (TE), which will go on to form extra-embryonic tissues (Rossant and Tam, 2009). Successful resolution of this first lineage decision is known to depend upon the activity of chromatin-modifying proteins (for recent reviews, see Burton and Torres-Padilla, 2014; Paul and Knott, 2014), but exactly how the activity of these key chromatin modifiers facilitates formation of specific cell lineages remains ill-defined.

CHD4 (chromodomain helicase DNA binding protein 4, also known as Mi-2β) is an ATP-dependent chromatin remodelling protein, first described as a component of the NuRD (nucleosome remodelling and deacetylation) complex in mammalian cells (Wade et al., 1998; Xue et al., 1998; Zhang et al., 1998). Mammalian CHD4 has been shown to be important for cell fate decisions in haematopoietic and epidermal development, but is dispensable for cell viability in both contexts (Hosokawa et al., 2013; Kashiwagi et al., 2007; Williams et al., 2004; Yoshida et al., 2008). Further roles for CHD4 have been described in embryonic angiogenesis (Curtis and Griffin, 2012), renal development (Denner and Rauchman, 2013), astroglial differentiation (Sparmann et al., 2013), neuronal maturation (Yamada et al., 2014) and in the control of cell cycle checkpoints and the DNA damage response (Larsen et al., 2010; O'Shaughnessy and Hendrich, 2013; Polo et al., 2010; Smeenk et al., 2010).

It has long been assumed that CHD4 and NuRD act to repress transcription, as NuRD contains histone deacetylase activity and has been shown to be capable of repressing transcription in a number of situations (Ahringer, 2000; McDonel et al., 2009). However, the advent of genome-wide technologies allows for a more global picture of protein behaviour in cells. Recent data show very clearly that NuRD, and CHD4, are not simply transcriptional repressors, but rather appear to modulate transcriptional output at numerous genes in a manner that is not yet fully understood (Gunther et al., 2013; Kim et al., 2014; Miccio et al., 2010; Reynolds et al., 2012a, 2013; Shimbo et al., 2013). In addition to its role in the NuRD complex, CHD4 has been reported to function independently of NuRD, often acting to promote gene expression (Amaya et al., 2013; Hosokawa et al., 2013; O'Shaughnessy and Hendrich, 2013; Williams et al., 2004). Chromatin immunoprecipitation followed by high-throughput sequencing (ChIP-Seq) in a variety of human cell lines or mouse tissues shows that CHD4 associates with the majority of promoters and enhancers in the mammalian genome, consistent with it being a general transcription co-factor (Gunther et al., 2013; Miccio et al., 2010; Morris et al., 2014; Reynolds et al., 2012b; Schwickert et al., 2014; Shimbo et al., 2013; Whyte et al., 2012; Zhang et al., 2012). Exactly how CHD4 influences transcription is not clear, although recently its activity has been associated with maintaining nucleosome density at some target sites (Morris et al., 2014; Moshkin et al., 2012).

Here, we use genetics and single-cell gene expression profiling to show that CHD4 is essential for successful resolution of the first lineage decision in mouse embryogenesis, and that this function is exerted independently of the NuRD complex. CHD4 is not required for cell viability in preimplantation stage embryos, but rather is required to restrict expression of lineage-specific genes. In the absence of CHD4 activity, outer cells are unable to sustain expression of appropriate lineage markers and fail to establish a TE fate. These data are consistent with a model in which control of gene expression by CHD4 defines the frequency at which a given cell will express lineage markers prior to a lineage-commitment event.

## RESULTS

### CHD4 function is required for blastocyst implantation

Mice heterozygous for a *Chd4* gene trap allele (RRO120; Bay Genomics; denoted *Chd4^−^*) or a *Chd4* interstitial deletion allele (denoted *Chd4*^Δ^) were viable and fertile, with no apparent heterozygote lethality (Table 1; for details of deletion allele, see supplementary material Fig. S1 and Materials and Methods), demonstrating that neither creates a dominant-negative allele. No phenotypic differences were detected between the *Chd4^−^* (RRO120) and the *Chd4*^Δ^ alleles. The majority of results presented here were generated using the RRO120 allele unless otherwise stated. Heterozygote intercrosses resulted in a 1:2 ratio of wild-type and heterozygote pups, but no *Chd4^−/−^* animals were found at weaning (Table 1). *Chd4^−/−^* blastocysts were readily recovered at 3.5 days post coitum (dpc), but none was recovered thereafter (Table 1). No increase in resorption sites or empty implantation sites was noted at early post-implantation stages, indicating that blastocysts lacking CHD4 fail to implant.

**Table 1.**
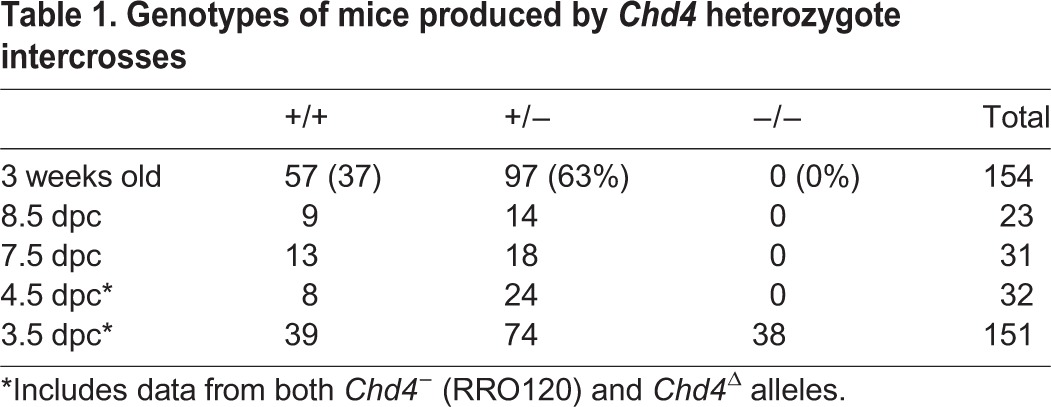
**Genotypes of mice produced by *Chd4* heterozygote intercrosses**

**Fig. 1.**
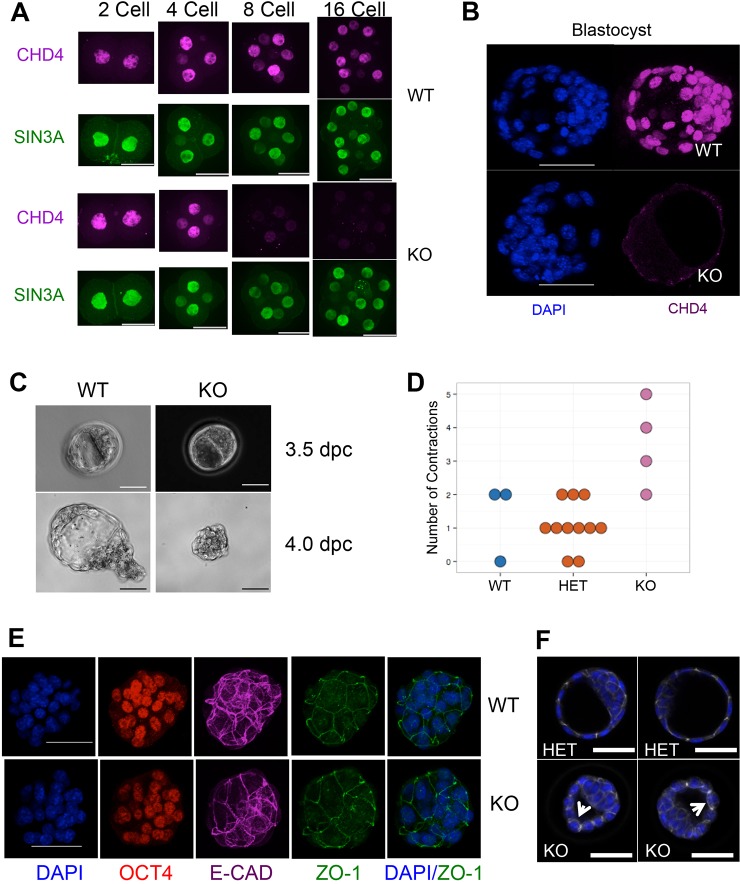
**CHD4 is required during the fourth day of development.** (A) Representative composite spinning-disc images of anti-CHD4 (magenta) and SIN3A (green; used as a control) staining in wild-type and *Chd4^−/−^* 2-, 4-, 8- and 16-cell embryos. Images are representative of eight null 2-cell embryos, 12 null 4-cell embryos, 11 null 8-cell embryos (including both *Chd4^−/−^* and *Chd4*^Δ*/*Δ^ embryos) and four null 16-cell embryos. Scale bars: 33 µm. (B) Composite confocal images of DAPI (blue) and CHD4 (magenta) staining in wild-type (WT) and *Chd4^−/−^* (KO) 3.5 dpc embryos. Scale bars: 50 µm. KO images are representative of >20 mutant blastocysts (including both *Chd4^−/−^* and *Chd4*^Δ*/*Δ^). (C) Phase contrast images of wild-type (WT) and *Chd4^−/−^* (KO) embryos flushed at different times on the fourth day post coitus (dpc). Scale bars: 50 µm. KO images are representative of 16 *Chd4^−/−^* embryos. (D) Average number of contractions per embryo genotype observed during live imaging (see supplementary material Movies 1-3). Each circle indicates the number of contractions for a single embryo of the indicated genotype. (E) Representative confocal 3D projection images of early 3.5 dpc embryos of the indicated genotypes stained for the indicated markers. KO images are  representative of six null embryos. Scale bars: 50 µm. (F) Two representative images per genotype of late 3.5 dpc embryos stained for E-cadherin (white) and DAPI (blue). Each image is a composite of four to six stacks, which allows visualisation of one entire cell layer across the distal end of the trophectoderm. Arrows indicate cells displaying mislocalised basal staining of E-cadherin. KO images are representative of eight null embryos. Scale bars: 50 µm.

CHD4 was present ubiquitously in cleavage-stage embryos (Fig. 1A). Nuclear CHD4 protein was detected at the 2-cell and 4-cell stages in both wild-type and *Chd4^−/−^* embryos, indicating that CHD4 protein either inherited from the oocyte or translated from maternally deposited mRNA was present in these early embryos (Fig. 1A). Null embryos at the 8-cell stage showed much reduced nuclear CHD4 staining, and staining was reduced to background levels in 16-cell mutant embryos. Similarly, ubiquitous nuclear CHD4 expression was detected in wild-type blastocysts, consistent with the X-gal staining seen in *Chd4* heterozygote blastocysts, but no protein was detected in null littermates produced from either allele (Fig. 1B; supplementary material Fig. S1B).

As *Chd4^−/−^* embryos could be recovered in Mendelian ratios at 3.5 dpc but were completely absent by 4.5 dpc (Table 1), we next undertook an analysis of the development of blastocysts during the fourth day of development. Blastocysts flushed in the morning of the fourth day (3.5 dpc) appeared morphologically normal and had formed a blastocoel (Fig. 1C). Late on the fourth day (4.0 dpc) wild-type embryos formed an expanded blastocyst in preparation for implantation, whereas mutant embryos collapsed into a tight ball of cells, with no evidence of a blastocoel. When cultured *in vitro*, *Chd4^−/−^* blastocysts were unable to attach and outgrow, even after removal of the zona pellucida (Table 2), indicating failure of trophectoderm function.

**Table 2.**
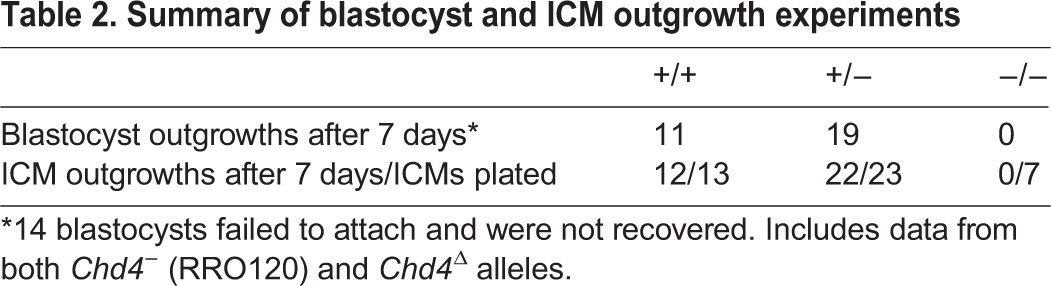
**Summary of blastocyst and ICM outgrowth experiments**

To visualise the embryonic failure of *Chd4* mutant embryos, the *ex vivo* development of morulae produced by *Chd4* heterozygote intercrosses was filmed in culture (supplementary material Movies 1-3). All morulae were able to give rise to cavitated blastocysts within the 48 h experiment. After blastocoel formation, wild-type and heterozygous embryos each showed between zero and two instances of blastocoel collapse, which were always followed by blastocoel re-expansion (supplementary material Movies 1-3; Fig. 1D). Mutant embryos similarly showed blastocoel collapse followed by re-expansion; however, this was followed by further collapses. Subsequent attempts to reform the blastocoel were decreasingly successful, and occasionally uncoordinated attempts could be seen occurring coincidently in the same embryo. Inevitably, mutant embryos failed to successfully establish a stable blastocoel and formed a collapsed structure similar to that flushed from uteri at 4.0 dpc (supplementary material Movies 1-3; Fig. 1C).

One possible explanation for a failure to maintain a blastocoel is that the integrity of the TE epithelium is not maintained in *Chd4^−/−^* blastocysts. Although we could find no evidence for a defect in either adherens or tight junctions in the trophectoderm of early *Chd4* mutant blastocysts by staining for E-cadherin (cadherin 1 − Mouse Genome Informatics) or ZO-1 (TJP1 − Mouse Genome Informatics) (Fig. 1E), later blastocysts contained cells in which E-cadherin was mislocalised basally in TE cells (Fig. 1F), consistent with a failure to properly maintain the polarity of the epithelial TE layer in *Chd4^−/−^* blastocysts (Fleming et al., 2001; Strumpf et al., 2005). As TE is required both for the maintenance of a blastocoel and for embryos to implant into the uterine wall, failure of TE formation or function would explain the precipitous loss of *Chd4^−/−^* embryos during implantation.

**Fig. 2.**
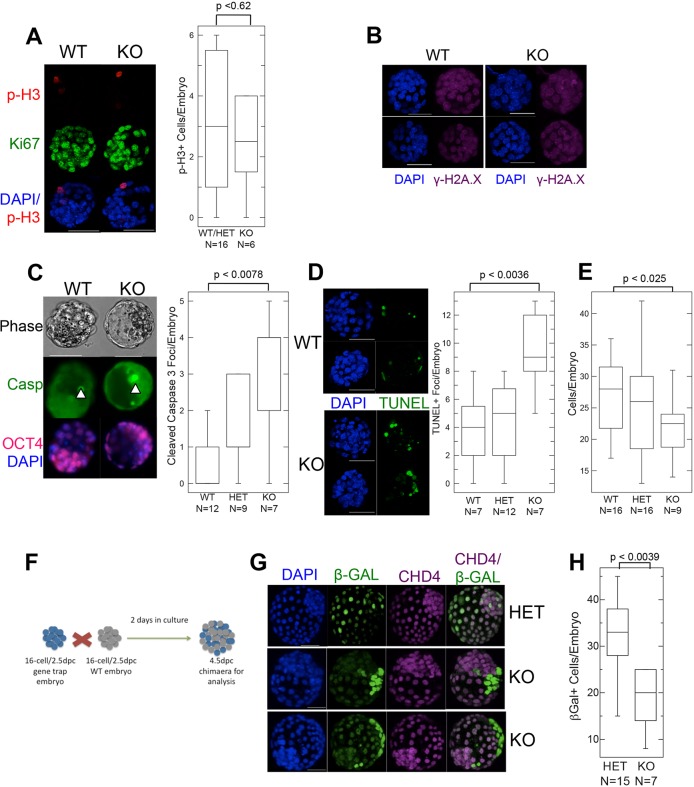
***Chd4^−/−^* embryos are viable before implantation.** (A) Left: Confocal 3D projections of 3.5 dpc embryos of the indicated genotypes stained for phosphorylated histone H3 (p-H3) and Ki67. Scale bars: 50 µm. Right: Quantification of the total number of p-H3-positive cells per embryo reveals no difference in wild type and heterozygous (WT/Het) versus knockout (KO) embryos. The numbers of embryos used to generate each box plot are indicated below the genotypes. (B) Confocal 3D projections of embryos of the indicated genotypes stained for γ-H2A.X (magenta) and DAPI (blue). KO images are representative of five null embryos. Scale bars: 50 µm. (C) Confocal images (left) of blastocysts of the indicated genotypes stained for cleaved caspase 3 (Casp; green), OCT4 (magenta) and DAPI (blue) scored for total cleaved caspase 3-positive cells per blastocyst (right). Arrowheads (left) indicate examples of positive cleaved caspase 3 staining. Scale bars: 50 µm. Numbers of embryos used to generate the box plots are indicated below each genotype. (D) Left: Confocal images of wild-type and *Chd4^−/−^* blastocysts assayed for TUNEL-positive cells (green). Scale bars: 50 µm. Right: Using confocal images, the total number of TUNEL-positive cells/embryo was calculated for each genotype. Numbers of embryos used to generate the box plots are indicated below each genotype. (E) Quantification of the average number of cells per embryo in early blastocysts of indicated genotypes. The numbers of embryos used to generate each box plot are indicated below the genotypes. (F) Schematic representation of aggregation experiments. Embryos from gene-trap litters (i.e. wild type, heterozygous or knockout) were aggregated to wild-type (WT) embryos. Heterozygous and knockout embryos contain the *lacZ* gene present in the gene trap allele, and hence express β-GAL (blue). (G) Confocal images of aggregation chimaeras, with genotypes of the test embryos, as inferred from anti-β-GAL (green) and anti-CHD4 (magenta) staining, indicated on the right. Scale bars: 50 µm. Images are representative of 11 different aggregations involving null embryos. (H) Plot of average number of cells from either heterozygous (HET) or *Chd4^−/−^* (KO) cells in chimaeric embryos. The numbers of embryos used to generate each box plot are indicated below the genotypes. All *P*-values were calculated using a two-tailed Mann–Whitney *U*-test.

The fact that morphologically normal *Chd4^−/−^* blastocysts were recovered early on the fourth day indicates that the cells were able to undergo at least two rounds of division in the absence of CHD4 protein, and therefore that CHD4 is not required for cell viability per se in cleavage stage embryos. This is in contrast to the situation in somatic cell lines, in which CHD4 has been shown to be important for cell cycle progression. Cells in *Chd4^−/−^* blastocysts stain positively for Ki67 (MKI67 − Mouse Genome Informatics) and show normal levels of phosphorylated histone H3 (Fig. 2A), a marker of mitotic cells, indicating that CHD4 is not required for cell cycle progression in blastocysts. Additionally, absence of CHD4 in blastocysts was not associated with notably increased levels of the histone variant γ-H2A.X, a marker of DNA damage (Fig. 2B), as was reported after CHD4 knockdown in somatic cells (Pegoraro et al., 2009). Nevertheless, *Chd4^−/−^* blastocysts show an increased proportion of apoptotic cells as measured by TUNEL or caspase staining (Fig. 2C,D) and, on average, contain a slightly reduced number of cells per embryo compared with wild-type or heterozygous littermates (Fig. 2E).

To evaluate further the viability of *Chd4^−/−^* cells, 16-cell embryos obtained from *Chd4* gene-trap heterozygote intercrosses were aggregated with wild-type 16-cell embryos and allowed to develop in culture for 48 h (Fig. 2F-H). Cells staining positively for both anti-β-GAL antibodies (indicating presence of the *Chd4* gene-trap allele) and for anti-CHD4 antibodies were deemed to be heterozygous, whereas β-GAL^+^, CHD4^−^ cells were considered to be *Chd4* null. *Chd4* null cells could be found contributing to morphologically normal chimaeric blastocysts after 48 h culture (Fig. 2G), demonstrating that *Chd4* is not required for cell viability in chimaeric blastocysts. It was notable that *Chd4^−/−^* cells failed to integrate with host cells, whereas *Chd4^+/−^* cells did, possibly indicating a difference in the physical properties of these cells. *Chd4^−/−^* morulae contributed about a third fewer cells to chimaeric blastocysts after 48 h culture than did *Chd4^+/−^* morulae (Fig. 2H), and these cells were more likely to stain positively for cleaved caspase 3 (7% of 69 null cells versus 0.3% of 160 heterozygous cells; not shown). Therefore, although CHD4 is not absolutely required for cell viability or cell cycle progression in preimplantation embryos, *Chd4^−/−^* cells show increased rates of apoptosis and may proliferate slower than heterozygous or wild-type cells.

### CHD4 is required for lineage specification in the early embryo

**Fig. 3.**
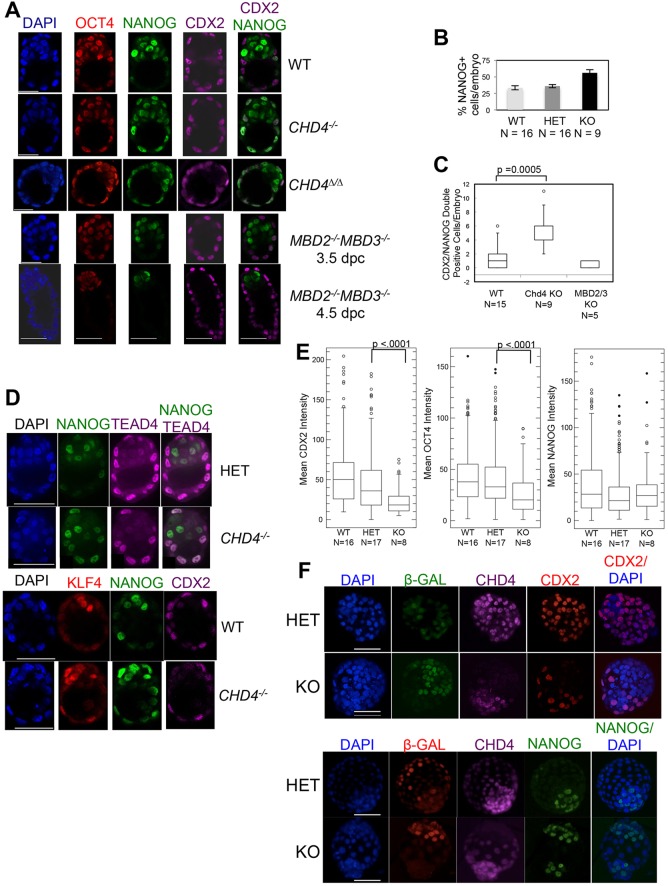
**Aberrant expression of lineage markers in *Chd4^−/−^* blastocysts.** (A) Confocal images (*z*-stack slice) of one wild-type (WT) blastocyst, one each of *Chd4^−/−^* and *Chd4*^Δ*/*Δ^ blastocysts, and one *Mbd2^−/−^Mbd3^−/−^* blastocyst, each at 3.5 dpc and 4.5 dpc stained for the markers indicated. Scale bars: 33 µm. Images are representative of 14 *Chd4* null blastocysts, five *Mbd2^−/−^Mbd3^−/−^* 3.5 dpc blastocysts and four *Mbd2^−/−^Mbd3^−/−^* 4.5 dpc blastocysts. (B) Plot of the average percentage of NANOG-expressing cells per blastocyst versus genotype, calculated by counting positive cells in whole embryos using 3D projections of the total number of *z*-stack slices taken. The number of embryos used to generate the data in this graph are indicated below the genotypes. Error bars represent s.e.m. (C) Plot of the number of cells in which both NANOG and CDX2 expression was detected by antibody staining in embryos of the indicated genotypes. Box plots were constructed using counts of the number of double-positive cells observed in one *z*-stack slice corresponding to the embryo mid-section. *P*-value was calculated using a two-tailed Mann−Whitney test. Embryo numbers are indicated. (D) One *z*-stack slice of a spinning-disc (top panels) or a confocal (bottom panels) image of embryos of the indicated genotype stained for the indicated markers. Scale bars: 50 µm. TEAD4 staining image is representative of seven null embryos; KLF4 staining image is representative of five null embryos. (E) Confocal images (3D projections of the total number of *z*-stack slices taken) were used to construct a box plot representation of mean intensity of individual CDX2-expressing (left), OCT-4-expressing (centre) and NANOG-expressing (right) cells versus genotype. Every cell was included in the analysis, i.e. no thresholding/exclusion of ‘non-expressing’ cells was necessary. *P*-values were calculated using a two-tailed Mann−Whitney test. Embryo numbers are indicated below the genotypes. (F) Confocal images of chimaeras produced from embryos of the indicated genotypes are shown stained for the indicated proteins. Images are representative of 11 different aggregations involving null embryos. Scale bars: 50 µm.

*Chd4^−/−^* embryos expressed CDX2 and TEAD4 in spatially appropriate patterns, in that nuclear localisation of both was detected exclusively or predominantly in outer cells, respectively (Fig. 3A-D). Note that OCT4 (POU5F1 − Mouse Genome Informatics) expression is not yet restricted to the ICM in these early blastocysts of either genotype. Although NANOG expression was predominantly localised to the ICM in both wild-type and mutant blastocysts, *Chd4* mutant blastocysts contained more NANOG-expressing cells than did wild-type or heterozygous littermates. This was largely due to aberrant expression of NANOG in outer cells, which also expressed the TE marker CDX2 (Fig. 3A-D). Cells co-expressing NANOG and CDX2 are infrequently observed in the trophectoderm of wild-type embryos (Strumpf et al., 2005); however, the *Chd4^−/−^* embryos displayed a notably increased proportion of NANOG/CDX2 double-positive cells (Fig. 3C). Some (but not all) NANOG/CDX2 double-positive cells were also found to express KLF4 in mutant embryos (Fig. 3D). By contrast, embryos lacking both *Mbd2* and *Mbd3*, which are expected to contain no functional NuRD complex, show a normal pattern of NANOG expression at both 3.5 dpc and at 4.5 dpc (Fig. 3A); this is 1 day later than *Chd4^−/−^* embryos can be recovered, providing evidence that this early requirement for CHD4 in lineage specification and embryo viability is exerted independently of the NuRD complex.

Quantitative analysis of staining intensity indicated that both CDX2 and OCT4 expression were reduced in cells of mutant blastocysts compared with wild-type or heterozygous littermates, whereas NANOG expression levels were unchanged (Fig. 3E). The lack of CDX2 expression was particularly notable in chimaeric embryos, in which, despite always being clustered in a peripheral location, *Chd4^−/−^* cells expressed low to undetectable levels of CDX2 (Fig. 3F). Instead, *Chd4^−/−^* cells, which were located in a position that would normally indicate a TE fate, either in null or chimaeric embryos, retained expression of the ICM marker NANOG (Fig. 3A,B,D,F). That this *Chd4^−/−^* phenotype occurs in the presence of wild-type cells in chimaeric embryos indicates that this is a cell-autonomous defect. It also demonstrates that *Chd4* null cells can survive beyond the point when lineage is normally specified, but do not show gene expression patterns consistent with successful TE formation.

### Failure to resolve cell fate-appropriate gene expression patterns in *Chd4*^−/−^ blastocysts

We next sought to determine whether and how CHD4 influences expression patterns of lineage-specific genes during this first lineage decision. To this end, we profiled gene expression in single cells isolated from preimplantation stage embryos by quantitative RT-PCR. A total of 56 different genes were chosen for expression analyses, including reference genes, developmental regulators and markers of pluripotent cells, TE, and primitive endoderm (supplementary material Table S1). Cells isolated from embryos at the 8- and 16-cell stages, and from early blastocysts, were subjected to directed cDNA amplification and qPCR. Overall, we analysed 33 and 35 cells each from wild-type/heterozygous and *Chd4^−/−^* 8-cell embryos, respectively; 37 and 46 cells from wild-type and null 16-cell embryos, respectively; and 59 and 62 cells from wild-type and null early blastocysts, respectively. For all stages, we took an average of five cells per embryo (although not all will have passed initial quality control, see Materials and Methods) and 7-16 embryos per condition. Our data showed a high degree of correlation with previously published single-cell gene expression data from these same time points using a similar RT-qPCR platform (Guo et al., 2010) (supplementary material Fig. S2).

**Fig. 4.**
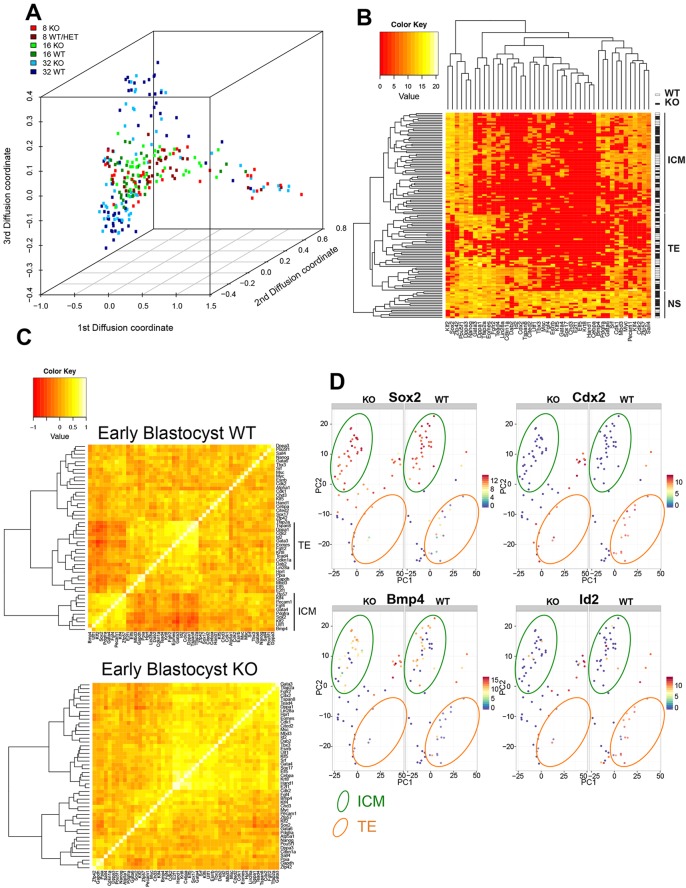
**CHD4 controls lineage-associated gene expression.** (A) Diffusion map of single cell expression data. Points represent individual cells of the indicated stages and genotypes. (B) Heat map produced from hierarchical clustering of early blastocyst single cell expression data. Individual cells are ordered from top to bottom, with a white box (on the right) indicating wild-type (WT) cells and a black box indicating *Chd4^−/−^* (KO) cells. Genes are listed across the bottom. Housekeeping genes (*Hprt*, *Atp5a1*, *Gapdh* and *Ppia*) were excluded from the comparisons to highlight the differences better. Cells cluster into three main groups, labelled ICM for inner cell mass cells, TE for trophectoderm cells, and NS for non-specified cells. (C) Heat map of pairwise Spearman correlations, generated using the Wards clustering algorithm (Murtagh and Legendre, 2014), between all genes used in the analysis in early blastocysts. Two major groups of correlated genes are visible in the wild-type plot (top), indicated as either ICM or TE genes on the right. (D) Principal component analysis (PCA) plots for early blastocyst single-cell expression data. Plots are shown in pairs, with data derived from mutant cells on the left (KO) and data from wild-type cells on the right (WT). Each filled circle represents one cell. Colours indicate the level of expression of *Sox2* (top left), *Cdx2* (top right), *Bmp4* (bottom left) or *Id2* (bottom right). The locations of cells displaying gene expression patterns characteristic of ICM or TE is indicated on the plot by green or orange ovals, respectively. Loadings for these PCA plots are shown in supplementary material Fig. S2D.

Displaying the expression data in a diffusion map (Coifman et al., 2005) allows for effective visualisation of the change in gene expression during developmental progression (Moignard et al., 2015) (Fig. 4A). Cells from 8-cell embryos display considerable heterogeneity in developmental timing, which is reflected in a wide distribution of 8-cell data points along the differentiation axis (Fig. 4A), but in general are highly similar to cells from 16-cell embryos. Cells from early blastocysts occupy largely the same space along the differentiation trajectory, but have segregated into two groups along the *z*-axis, corresponding generally to ICM and TE fates (data not shown). Comparing wild-type cells with mutant cells shows that loss of CHD4 does not dramatically change the transcriptional landscape of early embryonic cells, as cells generally cluster by stage of embryo rather than by genotype. This also shows that CHD4 mutant cells are unlikely to be developmentally delayed, because cells from *Chd4^−/−^* embryos cluster with wild-type cells from embryos of the same stage, rather than with cells from an earlier stage embryo (Fig. 4A).

At the early blastocyst stage, most wild-type cells have specified an ICM or TE fate, and unsupervised hierarchical clustering of the early blastocyst gene expression data shows that they cluster correspondingly into two main branches based upon gene expression (Fig. 4B). Wild-type cells cluster relatively evenly into ICM or TE fates (47.5% and 44.1%, respectively), whereas mutant cells are less likely to have adopted a TE fate (51.6% ICM versus 30.6% TE; supplementary material Fig. S3), consistent with a failure of TE formation in mutant blastocysts. A third, outlying cluster of cells is also visible in this plot, in which mutant cells outnumber wild-type cells by 2:1 (8.5% of wild-type cells, versus 17.7% of mutant cells). These cells express markers from both ICM and TE lineages, and are therefore labelled ‘NS’ for ‘non-specified’. The fact that this cluster contains some wild-type cells indicates that wild-type embryos do contain some non-specified cells at this early blastocyst stage; however, at this same stage null cells are twice as likely to remain unspecified.

At this stage, coordinated expression of genes associated with the ICM or TE lineages is apparent in wild-type cells, as is anti-correlation between these gene sets (Fig. 4C, top): the average correlation score among genes on the branch labelled ‘TE’ is 0.434±0.017, among genes on the ‘ICM’ branch is 0.416±0.021, and between ‘TE’ and ‘ICM’ genes is −0.138±0.015 (average±s.e.m.). By contrast, although *Chd4^−/−^* cells retain positive correlations among ICM and TE genes, they show a lack of anti-correlation between TE and ICM genes (correlation scores: TE-TE 0.420±0.015, ICM-ICM 0.365±0.018 and TE-ICM 0.151±0.017; Fig. 4C). Thus, cells in *Chd4^−/−^* embryos continue to express multiple lineage markers even at the blastocyst stage, and fail to establish exclusive lineage-appropriate gene expression programmes.

The distinction between ICM and TE cells in early blastocysts can be visualised in a principal component analysis plot (PCA plot; loadings shown in supplementary material Fig. S2D). Each spot represents a cell, and its location on the graph represents its total gene expression profile: cells showing TE-specific gene expression cluster towards the bottom right of the plot, and ICM-specific gene expression drives cells to the upper left (Fig. 4D). Both wild-type and mutant cells can be found displaying gene expression profiles consistent with an ICM cell fate (top left of PCA plots, Fig. 4D), providing further evidence that loss of CHD4 does not lead to widespread transcriptional dysregulation. The PCA plot of data from wild-type cells also shows a cluster of cells in the bottom right of the plot expressing TE markers, such as *Cdx2* and *Id2*, but in which expression of ICM markers, such as *Sox2* and *Bmp4*, is low or absent (Fig. 4D), consistent with a TE identity. Plots created from the mutant cells contain few cells in this TE region, but rather contain cells located between the TE and ICM areas, displaying expression of both TE and ICM markers, and these cells are predominantly contained within the outlier group in Fig. 4B labelled ‘NS’. Thus, CHD4 is important for blastocyst cells to maintain a gene expression profile consistent with a TE fate, but is not necessary for cells to exhibit an ICM gene expression programme.

### CHD4 limits gene expression frequency prior to lineage commitment

**Fig. 5.**
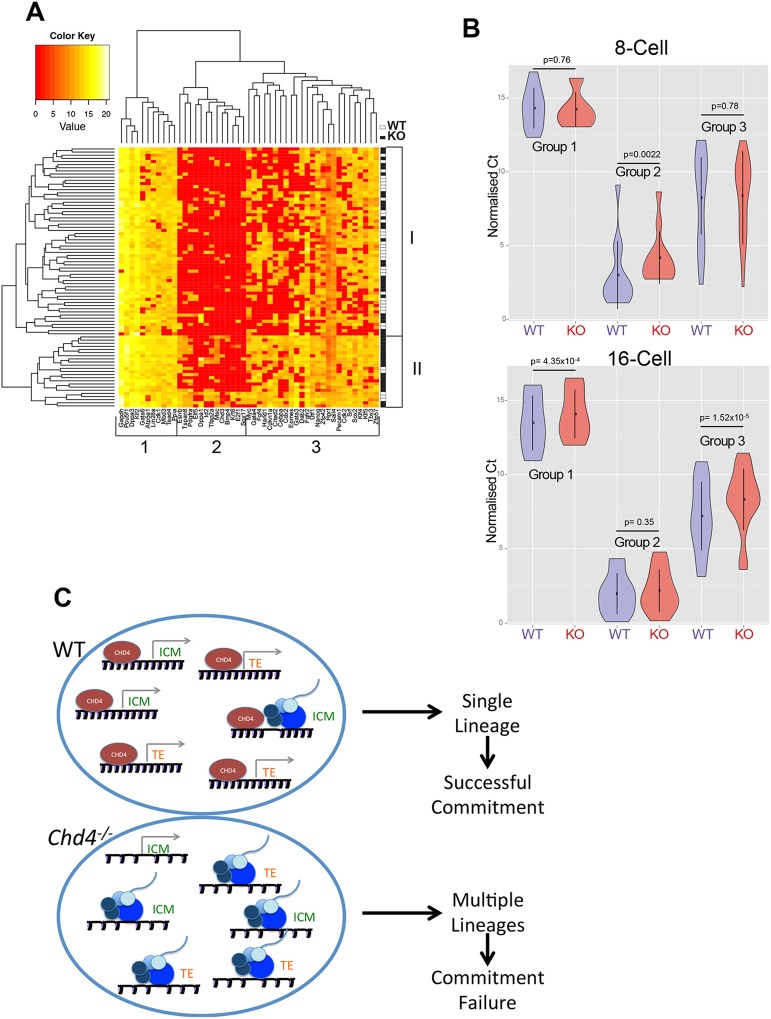
**CHD4 limits gene expression prior to lineage commitment.** (A) Heat map produced from hierarchical clustering of 16-cell expression data. Individual cells are listed from top to bottom, with a white box on the right indicating a wild-type (WT) cell and a black box indicating a *Chd4^−/−^* (KO) cell. Genes are listed across the bottom. The clustering algorithm produces two main groups of cells, labelled I and II, and three main groups of genes, labelled 1, 2 and 3. (B) Violin plots of expression data of Group 1, Group 2 and Group 3 genes (as shown in A) in wild-type (WT; blue) or mutant (KO; red) cells isolated from 8-cell (top) or 16-cell (bottom) embryos. The spot shows the mean and the vertical line shows the standard deviation. All *P*-values were calculated using a two-tailed Mann−Whitney test. (C) Model of how CHD4 is proposed to facilitate lineage commitment. Each large oval represents a cell, and the promoters for different lineage-specification genes are pictured. The genes pictured are either associated with a TE or ICM fate. A grey arrow indicates that the promoter is in a silent conformation, whereas the presence of RNA polymerase machinery (blue circles) and nascent RNA indicates an active conformation. In the wild-type situation (top), CHD4 acts to maintain nucleosome density across the promoters, such that binding by RNA polymerase is an infrequent event. In the absence of CHD4 (bottom), nucleosomes are positioned less densely, and transcription initiation is a much more frequent event. This results in the cell attempting to activate gene expression programmes for multiple lineages, and ultimately unsuccessful TE lineage commitment.

We next looked at 16-cell embryo data to see what influence CHD4 has upon gene expression prior to TE specification. Hierarchical clustering of the 16-cell expression data splits the genes assayed into three broad groups (Fig. 5A). Genes in Group 1, including housekeeping genes, *Pou5f1*, *Gata6* and *Tead4*, are expressed in nearly all cells, whereas Group 2 genes include later lineage markers, such as *Sox17*, *Dppa1* and *Esrrb*, and are expressed only rarely in 16-cell embryos. Group 3 genes include lineage markers, such as *Cdx2*, *Gata4*, *Sox2* and *Klf4*, but show a less consistent expression pattern than do Group 1 or 2 genes, in that they are expressed in some cells but not others (Fig. 5A).

Hierarchical clustering also splits the cells broadly into two classes, labelled Class I and Class II in Fig. 5A. Although both classes contain cells from both wild-type and mutant embryos, Class II comprises predominantly null cells (18 null cells versus five wild-type cells). These two classes differ in the frequency of expression of Group 3 genes: Class II cells express these genes at a higher frequency than do Class I cells, with the consequence that they are much more likely to show simultaneous expression of multiple lineage markers (Fig. 5A).

Comparing expression of the three different groups of genes between wild-type and mutant cells using violin plots (Fig. 5B) shows that at the 16-cell stage *Chd4^−/−^* cells tend to express slightly elevated levels of Group 1 (active) genes compared with wild-type cells (Fig. 5B). Although Group 2 genes show elevated expression in mutant 8-cell embryos, by the 16-cell stage both mutant and wild-type cells show a similar lack of expression of these genes (Fig. 5B), demonstrating that CHD4 is not globally required for gene silencing. The most pronounced difference between wild-type and mutant cells in 16-cell embryos is in the expression of Group 3 (variable) genes (Fig. 5B). Mutant cells from 16-cell embryos are more likely to express Group 3 genes than are wild-type cells, and they tend to express these genes at a higher level than do wild-type cells. This mis-expression is not seen at the 8-cell stage, when these genes show very heterogeneous expression in both wild-type and mutant embryos. Together, these data show that CHD4 functions to reduce the frequency of multi-lineage gene expression in 16-cell embryos, and that this ability correlates with subsequent successful resolution of the first lineage decision.

## DISCUSSION

Execution of the first lineage decision in mammalian embryogenesis requires that cells in the preimplantation stage embryo alter their gene expression programmes from that found in a totipotent cell to those defining either a pluripotent (e.g. ICM) cell or a multipotent (TE) cell. Here, we provide, at single-cell resolution, a temporal and molecular picture of how a chromatin remodelling protein influences transcriptional patterns during the first lineage commitment step in mammalian embryogenesis. These data are consistent with a model in which CHD4, a chromatin remodelling protein, facilitates cell fate specification by restricting the frequency of lineage-specific gene expression prior to specification in a cell-autonomous manner (Fig. 5D).

The observation that mutant embryos both fail to form a functional TE layer and fail to contribute to either lineage in chimaeric embryos is inconsistent with mutant embryos simply being developmentally delayed compared with wild-type littermates, but rather indicates a general failure to successfully maintain the TE lineage in *Chd4^−/−^* blastocysts. Nevertheless, some TE marker proteins are detectable in the outer cells of early *Chd4^−/−^* blastocysts, such as CDX2 (Fig. 3A). This indicates that aberrant gene expression at the 16-cell stage does not prevent the initial *Cdx2*-independent segregation of ‘outer’ versus ‘inner’ cells (Ralston et al., 2010; Ralston and Rossant, 2008). However, in the absence of CHD4, TE lineage specification is either incomplete or not maintained, leading to ‘outer’ cells transcribing both lineage-appropriate and -inappropriate genes (Fig. 4C). Although CHD4 has been shown to associate extensively with transcriptionally active chromatin in various cell types, we do not find evidence for global transcriptional dysregulation in preimplantation stage embryos lacking CHD4 (Figs 4 and 5). Rather, loss of CHD4 results in relatively subtle changes in gene expression at these early embryonic stages, which nevertheless culminates in embryonic failure.

Outer cells in *Chd4* null embryos appear to face a similar fate to those in *Cdx2* null embryos, in that they fail to maintain a blastocoel, they misexpress NANOG and they display an increased incidence of programmed cell death (Strumpf et al., 2005). ICM cells from *Cdx2^−/−^* embryos can give rise to embryonic stem cell (ESC) cultures (Ralston and Rossant, 2008; Strumpf et al., 2005), whereas *Chd4^−/−^* cells are lost prior to the stage at which ESCs arise (Boroviak et al., 2014), are unable to give rise to ESC cultures and are unable to contribute to the ICM in chimaeric embryos (Fig. 1C,E; Fig. 3F; Table 2). It is not immediately clear why ICM cells are unable to proliferate in the absence of CHD4, given that cells from cleavage stage embryos are able to survive for at least two cell divisions without CHD4. It is possible that the function of CHD4 in cell cycle progression described for somatic cells may become essential shortly after implantation, thereby precluding the viability of *Chd4^−/−^* epiblast cells.

Although CHD4 was the founding protein of the NuRD complex, our data support the existence of a NuRD-independent CHD4 function. *Mbd2*/*Mbd3* double-null embryos are able to form a compacted blastocyst with appropriate segregation of ICM and TE markers, providing strong genetic evidence that the role described here for CHD4 in resolving the first lineage decision is exerted independently of the NuRD complex (Fig. 3A). Biochemically, CHD4 has the ability both to move nucleosomes along the DNA and to displace nucleosomes from chromatin, and does not require other NuRD components for this activity (Wang and Zhang, 2001). Thus, caution should be exerted when interpreting experiments involving CHD4, as it is not necessarily an indicator of NuRD presence or activity as has commonly been assumed.

These data provide insight into how the first lineage commitment event in mammalian embryogenesis occurs. We show that cells present in 8- and 16-cell embryos variably express a number of markers of later lineages, as was also seen in a previous study (Guo et al., 2010). The lack of coordinated gene expression is inconsistent with a model of global sampling of lineage programmes, but consistent with stochastic activation of lineage-specific transcription factors prior to lineage commitment. Analogous situations have been described during haematopoiesis and neurogenesis, when stochastic or cyclic activation of key regulatory factors underlies the probability that a cell will enter a differentiation pathway (Imayoshi et al., 2013; Pina et al., 2012). This is also similar to events preceding the segregation of the epiblast and primitive endoderm lineages in late stage blastocysts, in which a period of seemingly random expression of epiblast and primitive endoderm markers in the ICM is followed by signal reinforcement and lineage segregation (Chazaud et al., 2006; Grabarek et al., 2012; Ohnishi et al., 2014; Plusa et al., 2008; Rossant and Tam, 2009).

In a developmental system in which stochastic expression of lineage markers occurs, the frequency at which these genes are expressed may be important in determining how the system develops over time. We propose that in 16-cell embryos CHD4 maintains the frequency of gene expression at a level that allows cells to enter into each lineage-specification pathway (although at this point we cannot prove that this change in gene expression frequency actually causes TE failure). CHD4 is a chromatin remodelling protein that can associate with the majority of promoters and enhancers in the mammalian genome, consistent with it being a general transcription co-factor, and has been shown to increase nucleosome density at its binding sites in both *Drosophila* and mammalian cells (Morris et al., 2014; Moshkin et al., 2012). Although at present we cannot test this in individual mutant morulae, we propose that normal function of CHD4 maintains nucleosome density across the promoters of lineage-specific genes, constraining probability of transcriptional firing (Lam et al., 2008; Raser and O'Shea, 2004) for each gene to a level consistent with successful commitment to a single lineage (Fig. 5C). In the absence of CHD4, nucleosome density would not be maintained, resulting in more accessible promoters and an increased probability of transcriptional activation (Fig. 5C). The observation that these embryos are subsequently unable to properly form the TE lineage is consistent with a model in which expression of multiple lineage-specification factors interferes with the ability of a cell to resolve a lineage-appropriate gene expression programme.

## MATERIALS AND METHODS

### ESCs, mice and embryos

All animal experiments were approved by the Animal Welfare and Ethical Review Body of the University of Cambridge and carried out under appropriate UK Home Office licenses. The *Chd4* gene-trap ESC line RRO120 was purchased from Bay Genomics. Sequencing of the trapped allele from the RRO120 ESC line revealed that the gene-trap cassette was inserted into exon 7 of the *Chd4* gene (supplementary material Fig. S1A). This cassette contains a splice acceptor sequence and a strong transcription termination signal, and therefore creates a fusion protein between the N-terminal *Chd4* exons and βgeo, which precludes translation of all exons located 3′ to the integration site. The *Chd4* conditional allele was created by introducing a LoxP site between *Chd4* exons 12 and 13; and a floxed drug selection marker between *Chd4* exons 21 and 22 by standard homologous recombination (supplementary material Fig. S1C). Transient expression of Cre recombinase was then used to remove the drug selection marker, leaving a single LoxP site between exons 21 and 22 and not affecting the LoxP site between exons 12 and 13. ESCs were cultured in standard ESC medium supplemented with 10% foetal bovine serum and recombinant mouse leukaemia inhibitory factor (LIF), or in 2i+LIF media (Ying et al., 2008) on gelatin-coated flasks.

ESCs were injected into C57Bl/6 host blastocysts to generate chimaeric mice using standard methods (Hogan et al., 1994). RRO120 *Chd4^+/−^* mice were bred onto the C57Bl/6 strain for at least ten generations. *Chd4^Flox/+^* mice were crossed to a line of mice expressing Sox2Cre (Hayashi et al., 2002) to facilitate deletion of the floxed *Chd4* allele, creating *Chd4*^Δ*/+*^ mice, which were then maintained by crossing to the C57Bl/6 strain. *Chd4^+/−^* mice were genotyped in a duplex PCR reaction using the following primers: 5′-AGGTCCCAATGCTCGGAGGAAGCC-3′, 5′-AGCCGAGTCACACCTGTCTGAAGC-3′; 5′-AGCGGATCTCAAACTCTCCTCCTTCCCTCC-3′. *Chd4*^Δ*/+*^ mice were genotyped in a duplex PCR reaction using the following primers: 5′-AGCATCTGGGAAGTTGTTGCTGCT-3′, 5′-TGACTGCCTGGAGAAAAGACACTCT-3′, 5′-TCCAGAAGAAGACGGCAGAT-3′. For embryo genotyping, an initial amplification of 22 cycles was carried out using the following primers: 5′-CCAGCTCATTTCCCATCATTTGCC-3′, 5′-TAGCTGTTGATACTGGCATCGTCG-3′, 5′-CCGGCGCTCTTACCAAAGGGCAAACC-3′ for the *Chd4*^–^ allele and 5′-GCCTCCTGAGTACTGGGATTAGGG-3′, 5′-GCTGCAACCACCCTTATCTCTTCC-3′, 5′-GAATTTGGCCCACAGCGAGAGT-3′ for the *Chd4^Δ^* allele.

Blastocyst outgrowths were performed using standard methods in standard ESC media on gelatin-coated plastic dishes. ICMs were isolated as described and outgrown in 2i/LIF ESC media (Hogan et al., 1994; Nichols et al., 2009). For live imaging, embryos were flushed using M2 medium (Sigma-Aldrich) at the 2.5-dpc stage. Each embryo was then placed in an individual well of an Embryo Immobilisation Chip and Interface (Dolomite Microfluidics) and cultured in KSOM (Millipore) using the Nikon Biostation (37°C, 5% CO_2_) for 48 h.

For aggregation experiments, 2.5-dpc RRO120 heterozygote intercross embryos were aggregated to 2.5-dpc wild-type embryos and cultured in KSOM until the 4.5-dpc stage. X-gal staining was performed as described (Kaji et al., 2006).

### Immunofluorescence

Embryos were flushed using M2 medium and the zona pellucida was removed if necessary using acid tyrodes solution. Embryos were then fixed in 2.5% paraformaldehyde, permeabilised in 0.25% Triton X-100 and 3% polyvinlypyrrolidone in PBS, and blocked in PBS containing 10% foetal calf serum and 0.1% Triton X-100. Primary antibodies were applied in blocking solution at 4°C overnight, and secondary antibodies for an hour at room temperature. Primary antibodies were used at the following dilutions in blocking solution: anti-CHD4 (1/200; 39289, Active Motif; and 1/10,000; ab70469, Abcam), anti-OCT4 (1/250; sc-5279 and sc-8628, Santa Cruz Biotechnologies), anti-NANOG (1/250; ab21603, Abcam), anti-CDX2 (1/250; Cdx2-88, BioGeneX; and 3977; Cell Signaling Technology), anti-cleaved CASPASE 3 (1/500; AF835, R&D Systems), anti-β-galactosidase (1/12,000; CGAL-45A, Immune Systems), anti-Ki67 (1/100; Clone SP6, Thermo Scientific), anti-phospho-H3 (S28) (1/150; H9908, Sigma-Aldrich), anti-E-cadherin (1/250; 610182, BD Biosciences), anti-ZO-1 (1/250; 402300, Life Technologies), anti-KLF4 (1/500; AF3158, R&D Systems) and anti-TEAD4 (1/500; ab58310; Abcam). Embryos were imaged in Ibidi μ-slides (Thistle Scientific). Confocal images, taken on a Leica SP5 confocal microscope, are 3D projections of the total number of *z*-stack slices taken, unless otherwise indicated. Spinning-disc images were taken on an Andor Revolution XD microscope. Confocal images were analysed using Fiji and Volocity software. For the quantification of levels of lineage markers, 3D projections of the total number of *z*-stack slices taken were analysed (i.e. whole embryos) and mean intensity values in each channel for every individual cell (assigned by Volocity software) were used to construct box plots (http://www.physics.csbsju.edu/stats/display.distribution.html). *P*-values were calculated using a two-tailed Mann–Whitney test (http://faculty.vassar.edu/lowry/utest.html).

For TUNEL staining, embryos had the zona pellucida removed and were fixed and permeabilised as above. They were then placed in TUNEL reaction mixture from the In Situ Cell Death Detection Kit, Fluorescein (Roche) for 60 min at 37°C, washed twice in PBS and DAPI-stained.

### Single-cell gene expression

Single cells were isolated from embryos by removal of the zona pellucida and subsequent dissociation in 1% trypsin in PBS with mechanical dissociation, using finely drawn glass capillaries. Some of the blastocysts were treated with Fluoresbrite YG Microspheres 0.20 µm (Polysciences) prior to dissociation as described (Burton et al., 2013). A selection of single cells was used for expression analysis, and the remainder of each embryo used for genotyping. Each single cell was placed in 9 µl of a pre-amplification mixture containing 0.05× of each TaqMan assay (supplementary material Table S1) (Life Technologies), 1× CellsDirect Reaction Mix (Life Technologies), 200 ng/µl Superscript III/Platinum Taq Mix (Life Technologies), 100 ng/µl SUPERase-In (Life Technologies) in DNA suspension buffer (TEKnova). The following program was then used for reverse transcription and specific target amplification in a thermal cycler: 50°C for 30 min, 95°C for 2 min followed by 24 cycles of 95°C for 15 s, 60°C for 4 min. cDNA was diluted 1:10 and loaded onto an OpenArray 56-assay/48 sample plate and the real-time PCR run on the Quantstudio 12 K Flex System with OpenArray block (Life Technologies). Only cells for which at least three of the four housekeeping genes showed expression were carried forward for analysis. The raw Ct values are provided in the supplementary material dataset.

All data manipulations and analyses were performed using R. The raw Cq values from the OpenArray were first transformed to Log2ex values then normalised to account for systematic biases. The Cq data were transformed to Log2ex by subtracting each data point from an empirically defined limit of detection (LOD=30), and then setting all negative values to zero. For each sample, the Log2ex data were normalised by subtracting the median Log2ex value for that sample from all of the Cq values, and then adding back the global median value. All negative values were set to zero. In an independent analysis, the Log2ex values were normalised to the mean of the housekeeping genes for that sample. Hierarchical clustering was performed using the gplots package using the function heatmap.2 and the hclust function. Principal component analysis was performed using the prcomp function. Housekeeping genes were removed from the PCA analyses for the early blastocyst data. Components were plotted using the ggplots package (Wickham, 2009). Diffusion maps were created using the diffusionMap package available in the CRAN R repository, and then plotted using scatterplot3d package available from CRAN. Correlations were performed using the cor function using the Spearman method. These correlations were plotted using gplots heatmap.2 function.

## Supplementary Material

Supplementary Material
