# Supplementary material for: Constraint of gene expression by the chromatin remodelling protein CHD4 facilitates lineage specification
Source: Development. 2015 Aug 1;142(15):2586–97. doi: 10.1242/dev.125450 (PMC4529036; doi:10.1242/dev.125450)
Supplement: Supplementary Material [file supp_142_15_2586__index.html]

Supplementary Material 

# Constraint of gene expression by the chromatin remodelling protein CHD4 facilitates lineage specification

## DEV125450 Supplementary Material

- Supplementary Material
