# Supplementary material for: Constraint of gene expression by the chromatin remodelling protein CHD4 facilitates lineage specification
Source: Development. 2015 Aug 1;142(15):2586–97. doi: 10.1242/dev.125450 (PMC4529036; doi:10.1242/dev.125450)
Supplement: Supplementary Material [file supp_dev.125450_DEV125450supp.pdf]

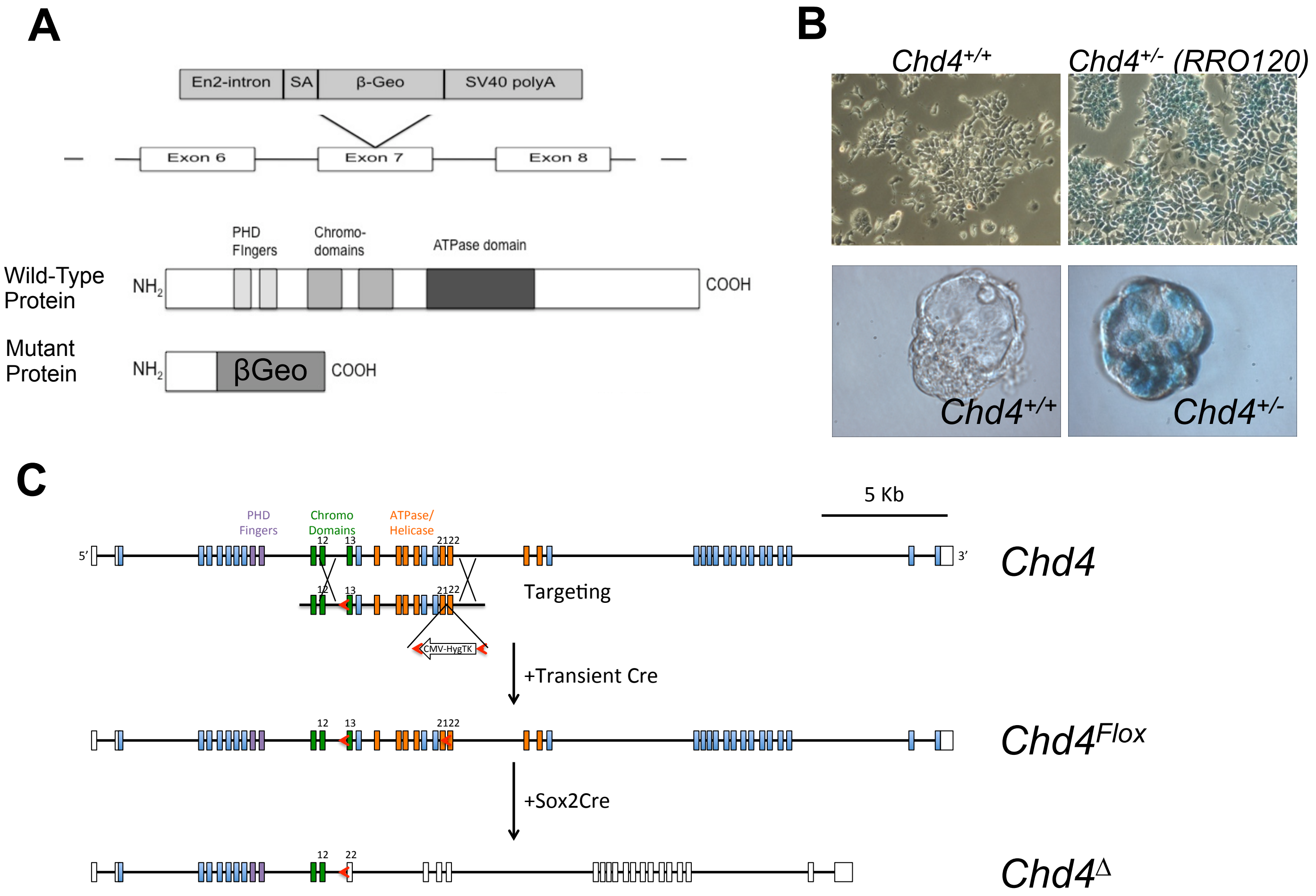

Supplemental Figure 1. *Chd4* mutant alleles
(A) Diagrammatic representation of the RRO120 *Chd4* gene-trap allele. Below are representations of the CHD4 wild-type protein and the truncated **b**-Geo-fusion protein expressed from the *Chd4* gene-trap locus. (B) X-Gal staining (blue) of wild-type and *Chd4*$^{+/-}$ ES cells (RRO120) (top) and blastocysts (bottom). (C) Diagrammatic representation of the creation of a conditional *Chd4* allele. A depiction of the *Chd4* gene is shown, with filled boxes representing coding exons and unfilled boxes representing non-coding exons. The targeting construct is shown below, containing one LoxP site (red arrow) between exons 12 and 13, and a floxed drug selection marker in the antisense orientation located between exons 21 and 22. After homologous recombination cells were treated with a low level of Cre recombinase to delete only the drug selection marker, resulting in a *Chd4*^{Flox} allele. These cells were used to make *Chd4*$^{Flox/+}$ mice, which were then crossed to mice expressing Cre under the Sox2 promoter (Hayashi, *et al*, 2002) to create the *Chd4*$^{\Delta}$ allele.

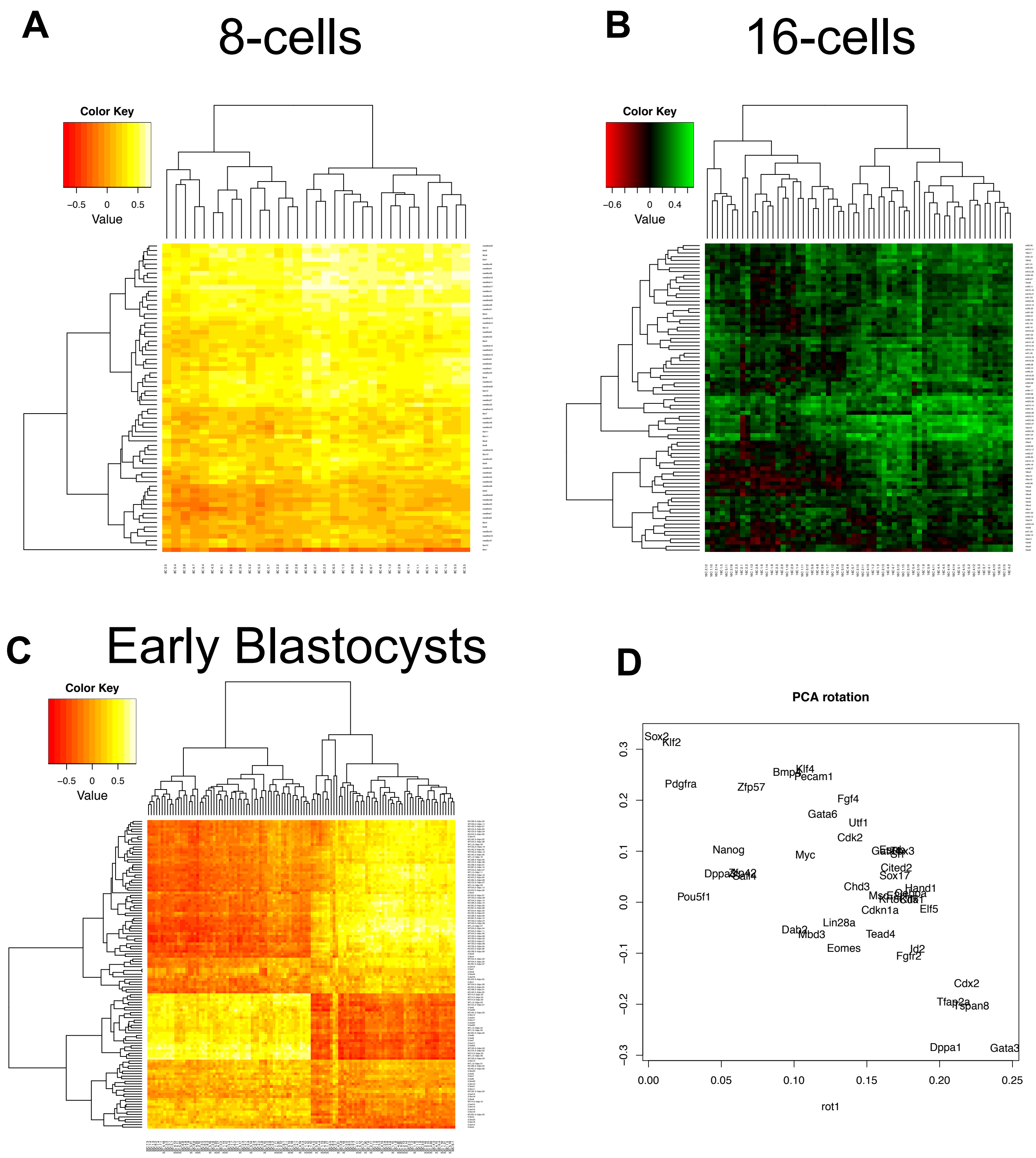

Supplemental Figure 2. Correlation plots of single cell gene expression data
Correlation plots comparing data described here with those presented in (Guo, *et al*, 2010) for (A) 8-cell embryos, (B) 16-cell embryos, and (C) early blastocysts. In (A) – (C) cells from this study are arranged vertically, and that from Guo et al. are listed across the horizontal. Good correlation exists in all comparisons. (D) Loadings for PCA plots in Fig. 4D.

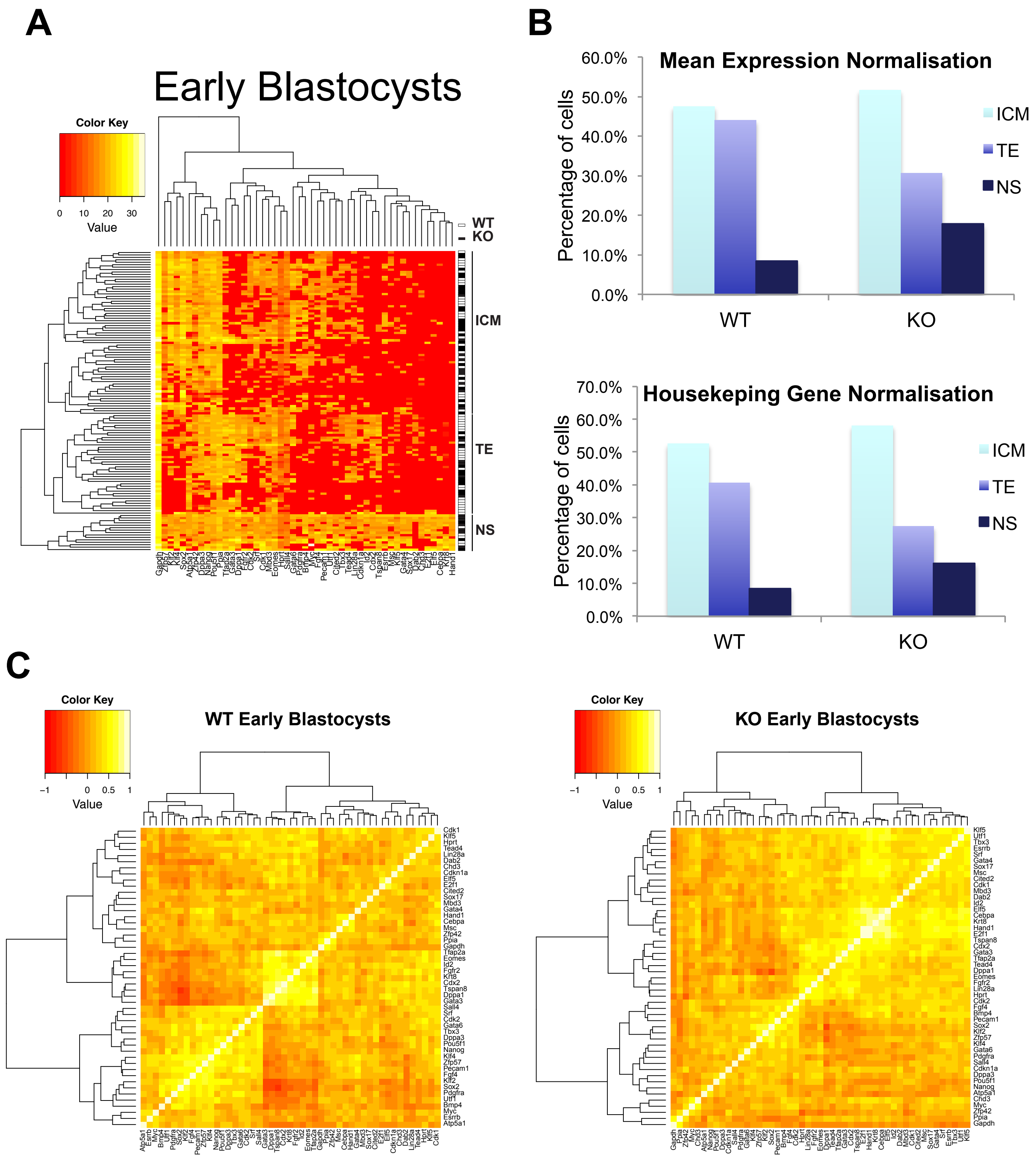
A
Early Blastocysts
Color Key
0 10 20 30
Value
WT
KO
ICM
TE
NS
B
Mean Expression Normalisation
Percentage of cells
60.0%
50.0%
40.0%
30.0%
20.0%
10.0%
0.0%
ICM
TE
NS
WT
KO
Housekeping Gene Normalisation
Percentage of cells
70.0%
60.0%
50.0%
40.0%
30.0%
20.0%
10.0%
0.0%
ICM
TE
NS
WT
KO
C
Color Key
−1 0 0.5 1
Value
WT Early Blastocysts
Color Key
−1 0 0.5 1
Value
KO Early Blastocysts

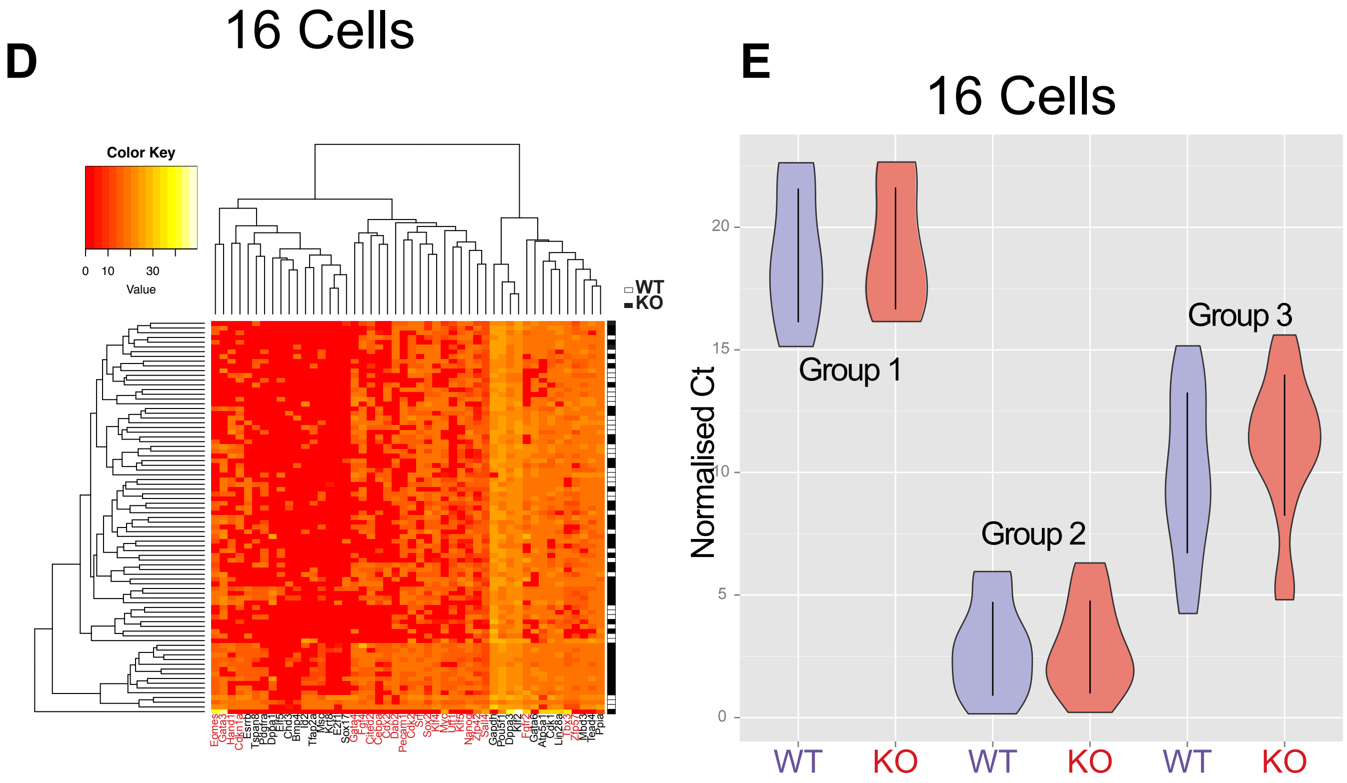

Supplemental Figure 3. Normalising single cell gene expression data (Figures 4 and 5) to housekeeping gene expression levels does not change data interpretation. The same analyses as shown in Figs. 4B, 4C, 5A and 5C are shown for data normalised to housekeeping gene expression in panels A, C, D, and E, respectively. Panel (B) plots the number of cells from the early blastocyst data which were clustered into the ICM, TE or NS categories after normalising data to mean expression (top) or housekeeping genes (bottom). In Panel D, those genes included in Group 3 (Fig. 5A) are indicated in red text. The two normalisation methods give very similar results.

Supplementary Movies

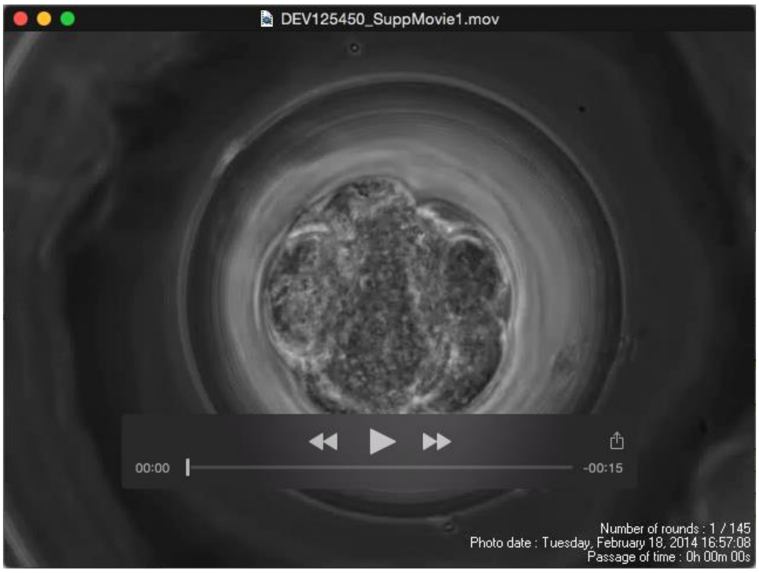

Supplementary Movie 1. Wild-type 16-cell embryo developing *in vitro* over 48 hours.

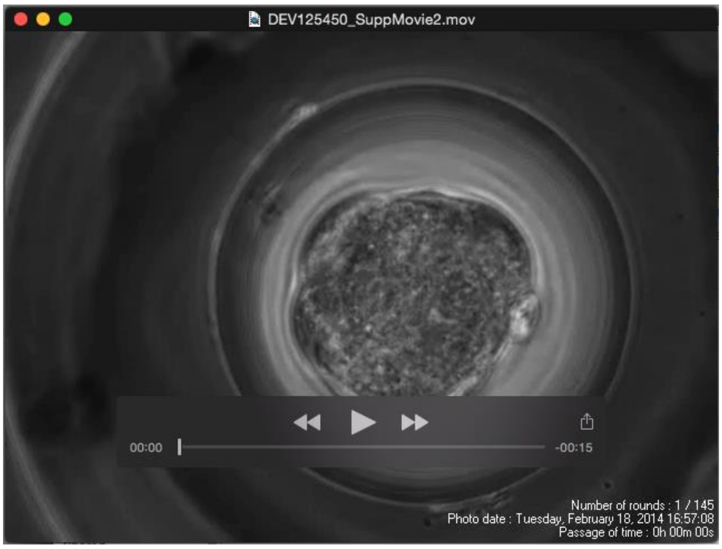

Supplementary Movie 2. *Chd4* heterozygous 16-cell embryo developing *in vitro* over 48 hours.

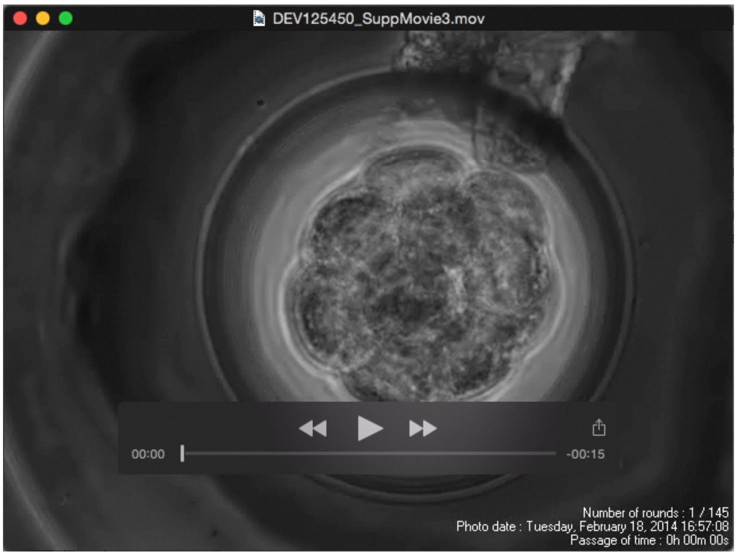

Supplementary Movie 3. *Chd4*$^{-/-}$ 16-cell embryo developing *in vitro* over 48 hours.

Table S1. Genes and assay IDs for OpenArray gene expression analysis

Gene	Assay ID
Atp5a1	Mm00431960_m1
Bmp4	Mm00432087_m1
Cdk1	Mm00772472_m1
Cdk2	Mm00443947_m1
Cdkn1a	Mm04205640_g1
Cdx2	Mm01212280_m1
Cebpa	Mm00514283_s1
Chd3	Mm01332658_m1
Chd4	Mm01190896_m1
Cited2	Mm00516121_m1
Dab2	Mm01307290_m1
Dppa1	Mm00626454_m1
Dppa3	Mm01184198_g1
E2f1	Mm00432936_m1
Elf5	Mm00468732_m1
Eomes	Mm01351985_m1
Esrrb	Mm00442411_m1
Fgf4	Mm00438917_m1
Fgf8	Mm00438922_m1
Fgfr2	Mm01269930_m1
Gapdh	Mm99999915_g1
Gata3	Mm00484683_m1
Gata4	Mm00484689_m1
Gata6	Mm00802636_m1

Hand1	Mm00433931_m1
Hprt	Mm01545399_m1
Id2	Mm00711781_m1
Klf2	Mm01244979_g1
Klf4	Mm00516104_m1
Klf5	Mm00456521_m1
Krt8	Mm04209403_g1
Lin28a	Mm00524077_m1
Mbd3	Mm00488961_m1
Msc	Mm00447887_m1
Myc	Mm00487804_m1
Nanog	Mm02384862_g1
Nr0b1	Mm00431729_m1
Pdgfra	Mm00440701_m1
Pecam1	Mm01242584_m1
Pou5f1	Mm03053917_g1
Ppia	Mm02342430_g1
Sall4	Mm00453037_s1
Sox17	Mm00488363_m1
Sox2	Mm03053810_s1
Srf	Mm00491032_m1
T	Mm01318252_m1
Tbx3	Mm01195726_m1
Tcf3	Mm01175588_m1
Tead4	Mm01189836_m1
Tfap2a	Mm00495574_m1
Tspan8	Mm00524563_m1

Utf1	Mm00447703_g1
Wnt4	Mm01194003_m1
Wnt5a	Mm00437347_m1
Zfp42	Mm03053975_g1
Zfp57	Mm00456405_m1

Table S2.

Click here to Download Table S2
